# Expression of the aquaglyceroporin HC‐9 in a freeze‐tolerant amphibian that accumulates glycerol seasonally

**DOI:** 10.14814/phy2.13331

**Published:** 2017-08-07

**Authors:** Brian Stogsdill, James Frisbie, Carissa M. Krane, David L. Goldstein

**Affiliations:** ^1^ Department of Biological Sciences Wright State University Dayton Ohio; ^2^ Department of Biology University of Dayton Dayton Ohio

**Keywords:** AQP9, Cope's gray treefrog, freeze tolerance, glycerol, liver

## Abstract

As ambient temperatures fall in the autumn, freeze‐tolerant Cope's gray treefrogs, *Dryophytes chrysoscelis* (formerly *Hyla chrysoscelis*), accumulate glycerol as a cryoprotective agent. We hypothesized that these treefrogs express an ortholog of the mammalian aquaglyceroporin AQP9 and that AQP9 expression is upregulated in the cold to facilitate glycerol transport. We sequenced 1790 bp from cloned cDNA that codes for a 315 amino acid protein, HC‐9, containing the predicted six transmembrane spanning domains, two Asn‐Pro‐Ala (NPA) motifs, and five amino acid residues characteristic of aquaglyceroporins. Functional characterization after heterologous expression of HC‐9 cRNA in *Xenopus laevis* oocytes indicated that HC‐9 facilitates glycerol and water permeability and is partially inhibited by 0.5 mmol/L phloretin or 0.3 mmol/L HgCl_2_. HC‐9 mRNA (qPCR) and protein (immunoblot) were expressed in most treefrog tissues analyzed (muscle, liver, bladder, stomach, kidney, dorsal skin, and ventral skin) except the protein fraction of red blood cells. Contrary to our prediction, both mRNA and protein expression were either unchanged or downregulated in most tissues in response to cold, freezing, and thawing. A notable exception to that pattern occurred in liver, where protein expression was significantly elevated in frozen (~4‐fold over warm) and thawed (~6‐fold over warm) conditions. Immunofluorescence labeling of HC‐9 protein revealed a signal that appeared to be localized to the plasma membrane of hepatocytes. Our results indicate that gray treefrogs express an AQP9‐like protein that facilitates glycerol permeability. Both the transcriptional and translational levels of HC‐9 change in response to thermal challenges, with a unique increase in liver during freezing and thawing.

## Introduction

While many organisms have developed the means to resist freezing in subzero temperatures, few vertebrates are freeze tolerant (Storey 1990). Freezing the body fluids can stress cells by introducing extracellular hypertonicity, cell shrinkage, ischemia, anoxia, and ice shearing (Costanzo et al. 1993). To support survival through these challenges, freeze‐tolerant animals accumulate cryoprotectants, which can include specialized antifreeze proteins as well as small metabolites such as glucose, urea, and glycerol (Costanzo et al. 2013).

In the North American wood frog, *Rana sylvatica*, the onset of freezing initiates the release of large quantities of glucose derived from glycogen stores in the liver, which increases the plasma glucose concentration within 5 min (Storey 1987). Other freeze‐tolerant frogs, such as *Dryophytes chrysoscelis* (formerly *Hyla chrysoscelis*), *Dryophytes versicolor* (formerly *Hyla versicolor*), *Pseudacris triseriata,* and *Pseudacris crucifer,* likewise liberate glucose during freezing (Storey and Storey 1986; Irwin and Lee 2003).

Besides glucose, *Dryophytes chrysoscelis* and its sister species *Dryophytes versicolor* also accumulate glycerol during cold acclimation prior to the onset of subfreezing temperatures (Layne and Jones 2001). The source of this glycerol is likely the metabolism of either hepatic glycogen or triglycerides stored in adipocytes (Irwin and Lee 2003). Glycerol concentrations increase in both extracellular and intracellular fluids (Storey and Storey 1986) and so glycerol must cross cell membranes for distribution and cellular uptake. At the same time, changes in glycerol permeability may contribute to regulating cell volume during freezing and thawing.

The primary mechanism by which glycerol crosses the plasma membrane is through transmembrane proteins belonging to the aquaporin family (Krane and Goldstein 2007). In mammals, thirteen aquaporin proteins (AQPs) contribute to plasma membrane water permeability. Of those, four are aquaglyceroporins (the GLPs AQP3, AQP7, AQP9, AQP10), which facilitate transport of glycerol in addition to water and may possess roles in metabolism, cellular tonicity, or cryopreservation (Mazur 1984; Madeira et al. 2015). As mammalian aquaporins lack any structurally obvious gating mechanism (Borgnia et al. 1999; Geijer et al. 2012), aquaporin‐mediated membrane permeability is likely to be mediated via changing protein expression in the cell membrane. In the short‐term, trafficking and sequestration of protein in intracellular vesicles may regulate localization of aquaporin proteins in the plasma membrane (Brown 2003). Over the longer term, transcription and translation of new protein may augment trafficking. Furthermore, modifications to the plasma membrane itself, as may occur during homeoviscous adaptation, can influence the function of aquaporins embedded in those membranes (Truniger and Boos 1993; Jensen et al. 2002; Hashido et al. 2005).

In mammals, hepatocytes express AQP9, which facilitates the uptake of glycerol used for gluconeogenesis, particularly during starvation (Jelen et al. 2011; Rodriguez et al. 2014). We hypothesized that *Dryophytes chrysoscelis* likewise expresses an AQP9‐like protein in liver, which could contribute to glycerol export to the circulation during cold acclimation (Zimmerman et al. 2007). Therefore, we predicted that AQP9 expression would increase during cold acclimation, freezing, and thawing as compared with warm‐acclimated conditions. To test this hypothesis, we sequenced an AQP9 homolog in *D. chrysoscelis*, hereafter referred to as HC‐9 in keeping with previously established nomenclature (Zimmerman et al. 2007), and characterized its function and expression in warm, cold, freezing, and thawing treefrogs.

## Materials and Methods

### Animal collection and maintenance

Male gray treefrogs, identified by trill frequency as *D. chrysoscelis*, were collected from ponds in Greene County, Ohio during the mating season (May–July). Animals were housed at Wright State University as approved by the Institutional Animal Care and Use Committee. Animals were held at 20°C, fed crickets, and given access to water for at least 2 months. In October, animals in the warm‐acclimated group remained at 20°C and a 12:12 (light: dark) light cycle, whereas the conditions for cold‐acclimated frogs were progressively shifted over a period of 2 months to 5°C with an 8:16 (light: dark) light cycle. Dishes of water were available to frogs in both groups. Cold‐acclimated frogs stopped eating when the temperature dropped to 10°C. Cold‐acclimated frogs remained at 5°C for 4–6 weeks prior to tissue collection while a subset were subjected to freezing by a further decrease in temperature from 5°C to −2.5°C over a 1‐week period. Freezing was then initiated by touching a piece of ice to the frog's dorsum. Ice formation proceeded for 24 h before sacrifice. Thawed animals were frozen for 24 h and then returned to 5°C and allowed to thaw for 24 h prior to sample collection.

### Tissue collection

Tissues were quickly removed from killed animals, flash frozen in liquid nitrogen, and stored at −80°C. Tissues were selected from a total of 20 animals from warm‐acclimated (*n* = 5), cold‐acclimated (*n* = 5), freezing (*n* = 5), and thawed (*n* = 5) conditions. The tissues used were collected between 2013 and 2015. Extracted and flash frozen tissue included muscle, liver, bladder, stomach, kidney, dorsal skin, ventral skin, small intestine, large intestine, brain, heart, fat, eye, lung, and red blood cells. A lobe of liver (*n* = 3) was also placed in fixative and embedded for IHC.

### RNA extraction

Total RNA was prepared from warm‐acclimated and cold‐acclimated *D. chrysoscelis* muscle, liver, bladder, stomach, kidney, dorsal skin, ventral skin, small intestine, large intestine, brain, heart, fat, eye, lung, and red blood cells using Tri‐reagent (Tri‐Reagent, Molecular Research Center, Cincinnati, OH) as previously described (Zimmerman et al. 2007). After a preliminary tissue panel, muscle, liver, bladder, stomach, kidney, dorsal skin, ventral skin, and red blood cells from warm, cold, frozen, and thawed animals (*n* = 5) were selected. These organs were selected based on the presence of HC‐9 signal in preliminary immunoblots (40 *μ*g of protein, 4% stacking/12% resolving gel using HC‐9 antibody described later), as well as their expected importance in the osmoregulation and metabolism of freeze tolerance. (e.g., muscular and hepatic role in glycerol storage, synthesis, and distribution, cutaneous regulation of water exchange, renal water, and glycerol regulation). RNA purity was determined by measuring the ratio of optical density at 260 nm/280 nm and total RNA integrity was confirmed with the absence of degraded RNA on a 1.5% agarose gel (in MOPS) run for 1 h.

### Sequencing HC‐9 and determining gene expression

#### cDNA synthesis

Total cDNA was generated with random hexamers using the Superscript III First‐Strand cDNA Synthesis kit (Invitrogen, Carlsbad, CA) from 1 *μ*g of total RNA in a 20 *μ*L reaction. Reactions without reverse transcriptase (–RT) were included as a control for DNA contamination of the RNA preparation.

#### Amplification of AQP9 mRNA using degenerate PCR

Amplification reactions consisted of 10 pmol of each primer, 0.25 mmol/L each dNTP, 1.5 mmol/L MgCl_2_, 60 mmol/L Tris‐HCl (pH 9.0), 12.5 mmol/L (NH_4_) 2SO_4_, 0.1 U of *Taq* DNA polymerase (New England Biolabs, Ipswich, MA), and 0.5 *μ*L of first‐strand cDNA from whole warm‐acclimated liver in a total reaction volume of 20 *μ*L. PCR was performed on an Eppendorf Mastercycler Gradient Thermocycler with 94°C for 3 min for an initial denature, 35 cycles at 94°C for 45 sec, 54° for 45 sec, 72° for 1 min, followed by a final elongation at 72° for 5 min using the degenerate primers dHC‐9 F 5′‐GCMCARTTYYTRGGWGCMWTT‐3′ and dHC‐9 R 5′‐YCKDGGWCYCARRKDGCWGGRTTYAT‐3′. PCR products were size‐fractionated on a 2% agarose gel in 1 × Tris‐Borate‐EDTA (TBE) at 100 volts for 2 h, stained with ethidium bromide, and visualized with UV light. The PCR products of the predicted size were extracted using the Qiagen QIAquick gel extraction kit (Qiagen, Valencia, CA), inserted into a plasmid vector, and sequenced. Gel‐extracted PCR products from degenerate primers were ligated into the plasmid cloning vector pGEM‐T‐Easy (Promega, Madison, WI), transformed into *Escherichia coli* strain JM109, and plated on LB‐ampicillin agar in the presence of 5‐bromo‐4‐chloro‐3‐indolyl‐*β*‐D‐galactopyranoside and isopropyl‐1‐*β*, D‐thiogalactopyranoside (Sigma‐Aldrich, St. Louis, MO) for blue‐white color selection toward the presence of an insert. Plasmid DNA was prepared using Miniprep spin columns (Qiagen, Valencia, CA) and restriction digested with *Eco*RI to excise the insert from the vector to confirm the presence of a ~350‐bp insert. The product was sequenced by Retrogen (San Diego, CA). Products with a high orthology to AQP9 were used as the basis for sequencing and primer design.

#### Sequencing and real‐time primers

18s RNA primers were developed from the partial sequence, Genbank accession #AF169014, to use as a housekeeping gene: 18s Forward 5′‐GTAGTAGCGGCAAGCAGTGT‐3′ and 18s Reverse 5′‐ATTCCCAGTAAGTGCGGGTC‐3′, which amplify a 76 bp fragment. Two internal HC‐9 primers were developed from the degenerate sequence and amplify an 81 bp fragment, HC9 Forward 3 5′‐ATGTCTACAGCTCTGCTGCTC‐3′ and HC‐9 3 Reverse 5′‐TGGCTCTAGTCCCTTTGGTG‐3′. Various primers were used to sequence the HC‐9 sequence out to the 5′ and 3′ ends, including HC‐9 Forward 8 5′‐GCACACAGAACAAACATTCCCA‐3′, HC‐9 Forward 10 5′‐ACACAGAACAAACATTCCCACG‐3′, HC‐9 Forward 11 5′‐AACCCAAAATCTGCAGAAGAGG‐3′, HC‐9 Reverse 6 5′‐TAACTGGGCCAAATGCTACAG‐3′, HC‐9 Reverse 8 5′‐TGTGCTCTGACATCTATGGGC‐3′, and HC‐9 Reverse 10 5′‐GCTTTTGTGTGCATAGGCCA‐3′.

#### Rapid amplification of cDNA ends (5′ and 3′ RACE)

5′ and 3′ RACE was performed using the primers developed from the previous partial sequence and two 5′/3′ RACE kits (Roche, Indianapolis, IN; Ambion, Foster City, CA) using cDNA from the liver of warm‐acclimated animals.

#### Sequence comparison

Full‐Length cDNA and the predicted amino acid sequence were submitted to GenBank with the accession number KU695572. Nucleotide and amino acid sequences were compared with previously published sequences available through GenBank using BLASTN and TBLASTX, respectively. The identity and query coverage of HC‐9 was compared with homologs from mammals and amphibians.

#### Quantitative real‐time reverse transcription PCR

The PCR amplification consisted of 10 pmol of each primer, 0.25 mmol/L each dNTP, 1.5 mmol/L MgCl_2_, 60 mmol/L Tris‐HCl (pH 9.0), 12.5 mmol/L (NH_4_) 2SO_4_, 0.1 U of *Taq* DNA polymerase (New England Biolabs, Ipswich, MA), and 1 *μ*L of cDNA (500 ng) in a total reaction volume of 20 *μ*L. cDNA from warm and cold‐acclimated animals was amplified using the primers HC9 Forward 3 and HC9 Reverse 3. PCR was performed on an Eppendorf Mastercycler gradient thermocycler with 94°C for 3 min for an initial denature, 35 cycles at 94°C for 30 sec, 58°C for 30 sec, 72°C for 30 sec, followed by a final elongation at 72°C for 5 min. The final product was size‐fractionated on a 2% agarose gel, stained with ethidium bromide, and imaged with UV light. The expected 81 bp fragment was identified, confirming the amplification of HC‐9. A –RT control was also gel fractionated, but did not show any fragments after a 35‐cycle amplification.

Using the same primers, relative expression levels of mRNA transcript for tissue from warm, cold, frozen, and thawed animals were determined by a qRT‐PCR run in triplicate, using 80 ng of cDNA per 20 *μ*L reaction, 10 *μ*L of 2 × SYBR Green, and 10 *μ*mol of each primer. 18s was used as a reference housekeeping gene. Each run included the presence of a warm‐acclimated tissue control, a negative water control, and the housekeeping reference run in tandem with the experimental group (cold‐acclimated, frozen, and thawed animal tissue‐extracted cDNA). PCR was performed on an Applied Biosystems 7900HT thermal cycler with an initial denature of 10 min at 95°C, followed by 40 cycles of 15 sec at 95°C, and 60 sec at 60°C. The 40th cycle was followed by a melt curve. Each melt curve had only one peak, suggesting the amplification of a single product.

The cycle at threshold (*C*_T_ value) was calculated using the standard curve settings in Applied Biosystems SDS 2.4. Relative gene expression was compared using the 2^−ΔΔ*C*T^ method (Greenwood et al. 2015). Expression of HC‐9 mRNA was normalized to expression of 18s rRNA and then quantified relative to the warm‐acclimated group.

### Protein expression and localization

#### Antibodies

An oligopeptide consisting of the C‐terminal sequence of 16 amino acids from HC‐9 (N‐EKHELANMTEKPKNRC‐C) was synthesized, and an antibody was generated against the KLH‐conjugated synthetic peptide in rabbits (Thermo Scientific, Rockland, IL). The antiserum was affinity purified using SulfoLink Immobilization Kit (Thermo Scientific, Rockford, IL), and the purified antibody was stored at −80°C until needed.

#### Protein isolation

A crude protein fraction was extracted in lysis buffer (50 mmol/L Tris‐HCl, 150 mmol/L NaCl, 1 mmol/L EDTA, 10% Glycerol, pH 7.4) with Protein Inhibitor Cocktail (PIC, Sigma‐Aldrich) added immediately before tissue homogenization as previously described (Pandey et al. 2010). Protein was extracted from muscle, liver, bladder, stomach, kidney, dorsal skin, ventral skin, and fat for warm‐acclimated, cold‐acclimated, frozen, and thawed (*n* = 3) animals, and from red blood cells for warm‐ and cold‐acclimated treefrogs.

#### SDS‐polyacrylamide gel electrophoresis and immunoblotting

Immunoblots were used to assess protein expression in muscle, liver, bladder, stomach, kidney, dorsal skin, ventral skin, and red blood cells. 40 *μ*g of protein was loaded on a 4% stacking/12% resolving gel. The gel was then blotted onto a PVDF membrane, blocked, incubated, and washed as previously described (Pandey et al. 2010). All uses of PBS and PBST were substituted with TBS and TBST. The primary antibody against HC‐9 was applied using a 1:500 dilution at a concentration of 0.38 *μ*g/*μ*L, whereas the secondary antibody (donkey anti‐rabbit IgG‐HRP; Santa Cruz, Dallas, TX) was incubated at a 1:5000 dilution. Immunoblots of protein expression in the cold‐acclimated, frozen, and thawed conditions were quantified by densitometry and normalized to *β*‐actin expression, then expressed relative to values for the warm‐acclimated condition (Fujifilm Multi Gauge V2.3).

#### Deglycosylation

Protein from warm‐acclimated, cold‐acclimated, frozen, and thawed liver was treated with N‐glycosidase F (PNGase F; New England BioLabs, Ipswich, MA) to assess the contribution of protein glycosylation to the presence of high‐molecular‐weight bands in immunoblots. 40 *μ*g of protein was mixed with denaturing buffer (0.5% SDS and 40 mmol/L DTT), then incubated in reaction buffer (50 mmol/L sodium phosphate buffer with 1% NP40, pH 7.5) with 1000 U of PNGase for 30 min at 37°C. The same quantity of protein from each tissue was subjected to the same conditions without the presence of the enzyme. The treated and untreated proteins were loaded on a 4% stacking/12% resolving SDS–polyacrylamide gel for electrophoresis for 1 h at 150 V and transferred to PVDF membrane for immunoblot analysis.

#### Histology and immunofluorescence

A lobe from warm‐acclimated and cold‐acclimated treefrog liver was quickly removed by dissection and immersed in periodate‐lysine‐paraformaldehyde (PLP; 4% paraformaldehyde, 75 mmol/L lysine, 37.5 mmol/L sodium periodate, and 10 mmol/L Na2HPO4, pH 7.2) overnight. Tissues were dehydrated, embedded in paraffin, cut into 10 *μ*mol/L sections, and placed on slides as previously described (Pandey et al. 2010). Liver sections were rinsed in PBS (3 × 5 min), followed by treatment with 0.2% TX‐100 (in PBS, pH 7.2), 1% Glycine (in PBS, pH 8), and 0.1% NaBH_4_ (in PBS, pH 8) for 15 min each. Sections were rinsed in PBS again (2 × 5 min), blocked (10% BSA, 10% Serum, 0.05% TWEEN® 20 in PBS, 0.1 mol/L, pH 7.2) for 1 h at room temperature and incubated overnight at 4°C in primary antibody (HC‐9, 1:200, in 1% BSA). After another wash in PBS (3 × 5 min), the section was incubated with 1:500 cy5‐conjugated goat anti‐rabbit secondary antibody in 1% BSA. DAPI was added to mounting medium (Santa Cruz, Dallas, TX). Z‐stack of images was taken using an Olympus FV1000 confocal microscope and edited using Olympus Fluoview Ver.1.7a.

### Functional characterization of HC‐9 in *Xenopus* oocytes

Oligonucleotide primers upstream of the predicted start codon and downstream of the stop codon of the HC‐9 cDNA sequence were used to generate a 1631 bp PCR product. The PCR product was amplified using Phusion High Fidelity DNA polymerase (Thermo Fisher Scientific, Waltham, MA), purified using the Roche high pure PCR purification kit (Roche, Indianapolis, IN), cloned into the pGEM–T–Easy vector (Promega, Madison, WI), transformed into DH5*α*‐T1 *Escherichia coli* competent cells (Thermo Fisher Scientific), and then plated on ampicillin‐treated LB agar. Colonies were selected and grown in LB broth with ampicillin, and then the plasmids were extracted with a Qiagen Miniprep (Qiagen, Valencia, CA). The HC‐9 plasmid was digested and a *Xenopus β*‐Globin 5′ Untranslated region (UTR) was inserted immediately upstream of the HC‐9 sequence to facilitate translation and increase HC expression in oocytes as described previously (Zimmerman et al. 2007). The *Xenopus β*‐globin 5′ UTR was PCR amplified from a *Xenopus* oocyte expression vector containing human AQP1 (hAQP1; American Type Culture Collection, Rockville, MD). The hAQP1 and the HC‐9 plasmids containing *Xenopus β*‐globin 5′ UTR were transformed, grown, and extracted as previously described.

Linearized DNA (*Sma*I digestion) from the HC‐9 plasmid was used as a template for in vitro transcription of complementary RNA (cRNA) in the presence of the 7‐methylguanosine cap using T7 polymerase (mMESSAGE mMACHINE, Ambion, Austin, Tx) and purified with MEGA CLEAR columns (Ambion) as previously described (Zimmerman et al. 2007). *Xenopus* oocytes were extracted from live animals, isolated with collagenase, and incubated at 18°C overnight in modified Barth's solution before injection (Carbrey et al. 2003; Zimmerman et al. 2007; Hirota et al. 2015). Oocytes (*n* = 10) were injected with 50 ng of cRNA coding for HC‐9, HC‐9/hAQP1 (dual injection of 25 ng each), hAQP1, or with 50 nL of water (negative control), and incubated at 18°C for 72 h in modified Barth's buffer which was replaced every 24 h. To confirm the presence of HC‐9, oocytes injected with HC‐9, hAQP1, and water (*n* = 5 of each condition) were pooled and digested after 72 h. Protein was extracted using protein lysis buffer and PIC, and 30 *μ*g of protein was electrophoresed for 1.5 h and immunoblotted as described previously.

To measure water permeability, oocytes were transferred to a cell culture dish containing modified Barth's solution at 20°C, diluted with water to 67 mosmol/kg H_2_O. Oocytes were photographed every 30 sec for 5 min or until they burst. To assess glycerol permeability, oocytes were placed in isosmotic modified Barth's solution in which 130 mmol/L glycerol replaced equiosmolar NaCl. To determine inhibitor sensitivity, some oocytes were preincubated for 5 min before hypotonic or glycerol challenge in either 0.3 mmol/L HgCl_2_ or 0.5 mmol/L phloretin, a known AQP9 inhibitor (Calamita et al. 2012). To determine the effect of cold temperature on permeability, some oocytes were preincubated at 10°C for 10 min before the hypotonic challenge, which was also carried out at 10°C. Oocyte areas were imaged on a Nikon Eclipse TE 2000‐S microscope with a Cool Snap ES camera using Metamorph version 6.1r4 software. ImageJ 1.34s software was used to measure area and radius, which was used to calculate volume. The nonisotopic solute permeability (*P*_s_) and the coefficient of osmotic water permeability (*P*_f_) of each condition was calculated from the oocyte surface area (*S* = 0.045 cm^2^), initial oocyte volume (*V*_o_ = 9 × 10^−4^ cm^3^), the initial slope of the relative volume increase (*V*/*V*_o_)/dt for 300 sec or until bursting, the total osmolality of the system (osm_total_ = 200 mosM), and the osmotic solute gradient (sol_out_−sol_in_) as follows: *P*_s_ = [osm_total_ × *V*_o_ × *d* (*V*/*V*_o_)/*dt*]/[*S* × (sol_out_−sol_in_)] (Carbrey et al. 2009) and *P*_f_ = [*V*_o_ × *d* (*V*/*V*_o_)/*dt*]/[*S *× *V*_w_ (osm_out_−osm_in_)] (Zhang et al. 1990).

### Statistics

All data are expressed as a mean ± standard error (SEM). Statistical differences between group means were analyzed using one‐way ANOVA followed by Tukey's Multiple Comparison test. All tests were performed with a 95% confidence interval, where *α *≤ 0.05 is considered significant. For studies of membrane permeability of oocytes, pairwise comparisons of *P*_f_ values between the HC‐9 clone and controls were evaluated using Student's two‐tailed t‐tests with equal variance, and influences from temperature and inhibitors were determined by ANOVA (Zimmerman et al. 2007). All statistical analyses were performed using PRISM 5.03 and Excel 2013.

## Results

### HC‐9, an AQP9 homolog from *Dryophytes chrysoscelis*

**Figure 1:**
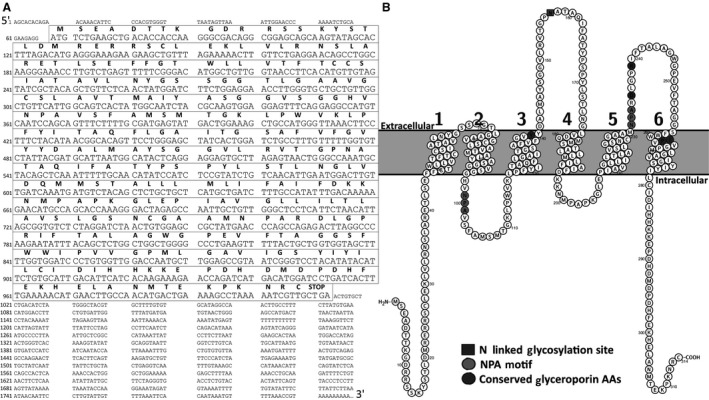
Nucleotide and Protein Sequence of *Dryophytes chrysoscelis HC‐9*: (A) The nucleotide sequence of HC‐9 from *D. chrysoscelis* is shown with the translated (boxed) and untranslated (unboxed) regions displayed. The predicted amino acid sequence is shown above the nucleotide sequence. (B) The protein secondary structure topography was predicted using Phobius and generated using Protter (http://wlab.ethz.ch/protter/). Includes the predicted location of the NPA motifs, the N‐linked glycosylation site, and five conserved aquaglyceroporin amino acids.

Several sequenced products from 5′ and 3′ RACE were assembled to create a 1790 bp sequence (HC‐9). The HC‐9 sequence includes a predicted 945 bp translated region (Fig. 1A). The start codon resides at position 68–70, and the stop codon resides at position 1010–1012. The sequence translates to a 315 amino acid protein with a calculated molecular mass of 33.69 kDa. A second possible start codon resides 60 nucleotides downstream of the first codon within the same reading frame. This would code for a 295 amino acid protein with a molecular mass of 31.45 kDa. The translated amino acid sequence includes two NPA (Asparagine‐Proline‐Alanine) motifs at residues 99–101 and 231–233 and conforms to a classic aquaporin configuration in the MIP family, including six transmembrane domains with two intracellular and three extracellular loops as predicted using the topology predictor Phobius (Fig. 1B). The sequence contains five amino acids (Y‐139, D‐235, R‐239, I‐261, P‐262) conserved in aquaglyceroporins (Hall et al. 2015; Fig. 1B). A possible N‐linked glycosylation site is found at position 157 on the second extracellular loop. Possible phosphorylation sites (score > 0.9) exist at S13, S14, S17, S26, T6, and T41, based on predictions using NetPhos 3.1 (http://www.cbs.dtu.dk/services/NetPhos/).

**Figure 2:**
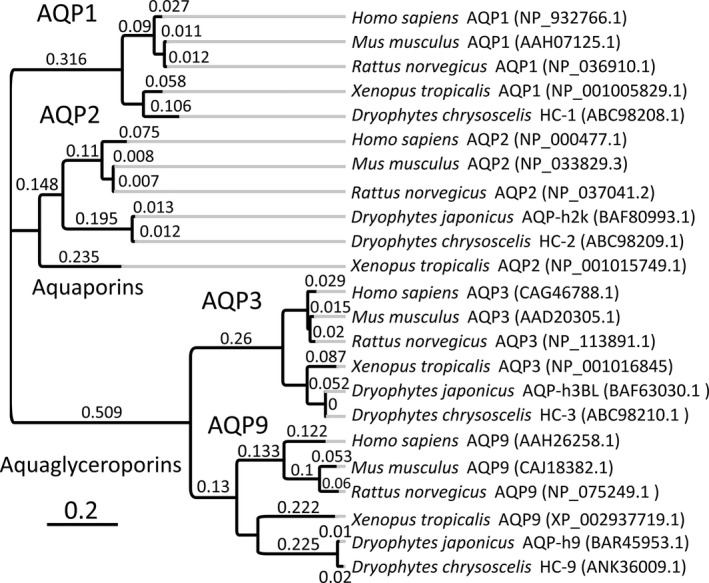
Comparison of AQP1, AQP2, AQP3, and AQP9 in *Dryophytes chrysoscelis* Based on the Amino Acid Sequence: An unrooted tree of aquaporins and aquaglyceroporins identifies all aquaporins currently published from the *D. chrysoscelis* (HC‐1, HC‐2, HC‐3, and HC‐9). The protein sequences from *Dryophytes chrysoscelis* are compared *to* the protein sequence of *Dryophytes japonicus* (Japanese Treefrog), *Xenopus laevis* (African Clawed Frog), *Xenopus tropicalis* (Western Clawed Frog), *Mus musculus* (house mouse), and *Homo sapiens* (humans). The scale represents genetic change as amino acid substitutions per site. The sequence is aligned and generated using Geneious version 10.0.9 (http://www.geneious.com, Kearse et al. 2012).

The nucleotide sequence is strongly homologous to AQP9, with 97% sequence identity to *Dryophytes japonicus* (formerly *Hyla japonica*) AQP‐h9 (LC008216.1) and 71% sequence identity to the predicted *Xenopus tropicalis* AQP9 (XM_002937673.3). The predicted translated protein sequence also shows high homology to AQP9 (Fig. 2), with 97% sequence identity to *Dryophytes japonicus* AQP‐h9 (BAR45953.1), 62% sequence identity to the predicted *Xenopus tropicalis* AQP9 *(*XP_002937719.1), and 59% sequence identity to *Homo sapien*s AQP9 (NP_066190.2).

### Tissue pattern of mRNA expression

Reverse transcription PCR using primers specific to HC‐9 yielded a single 81 bp band that was present in muscle, liver, bladder, stomach, kidney, dorsal skin, ventral skin, small intestine, large intestine, brain, heart, fat, eye, lung, and red blood cells from both warm‐ and cold‐acclimated treefrogs (not shown).

**Figure 3:**
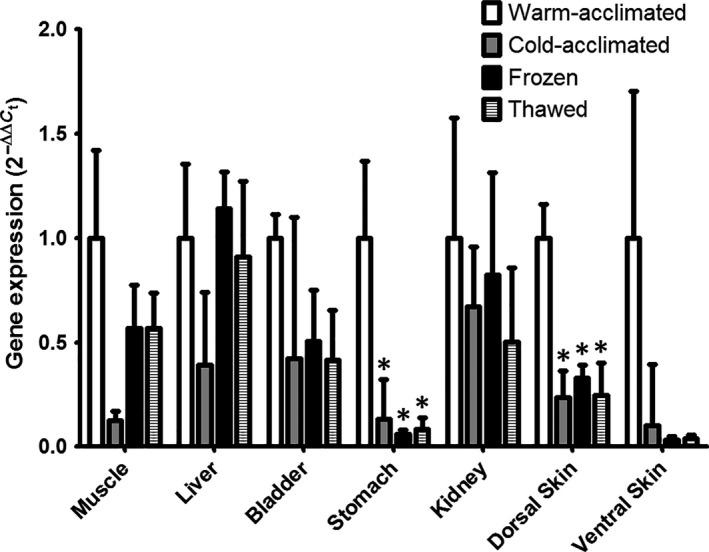
Comparative Real‐time Expression of HC‐9 mRNA in the *Dryophytes chrysoscelis*: HC‐9 mRNA expression is shown for muscle, liver, bladder, stomach, kidney, dorsal skin, and ventral skin of the *D. chrysoscelis*. Real‐time PCR of selected tissues from warm‐acclimated, cold‐acclimated, frozen, and thawed treefrog tissue were measured (*n* = 5). The standard mean of 2^−ΔΔCt^ is graphed with ±SEM. * indicates a condition with a *P* < 0.05 compared by one‐way ANOVA to data from the warm‐acclimated condition.

Quantitative PCR was performed on the selected tissues muscle, liver, bladder, stomach, kidney, dorsal skin, and ventral skin. The result revealed a significant difference in relative mRNA expression between warm, cold, frozen, and thawed tissue in the stomach [*F* (3, 16) = 4.691, *P* ≤ 0.0155] and the dorsal skin [*F* (3, 16) = 6.156, *P* ≤ 0.0069] (Fig. 3). In both tissues, expression was diminished in all colder conditions: 75% and 72% reduction in stomach and skin, respectively, from cold‐acclimated animals; 93% and 72% reduction in tissues from frozen animals; and 90% and 67% reduction in tissues from thawed animals.

### Expression of HC‐9 protein

**Figure 4:**
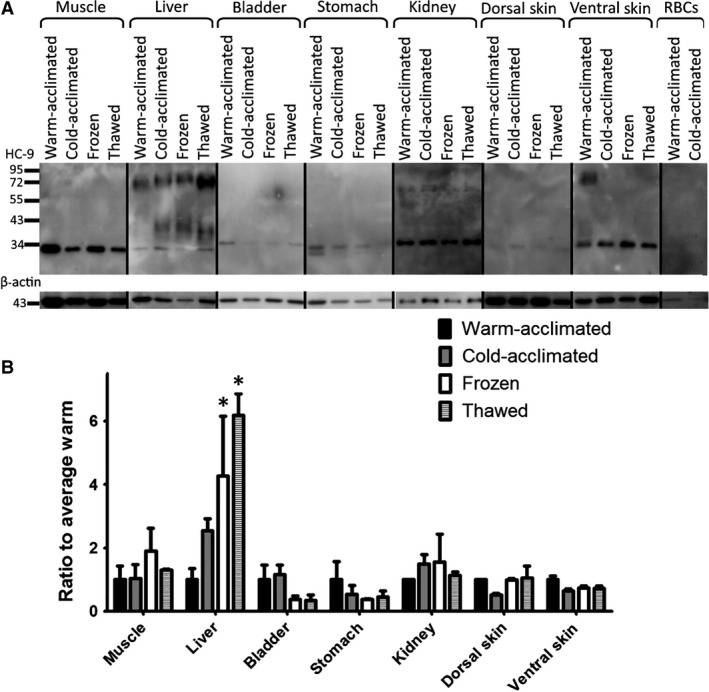
Protein Expression by Immunoblot for Various Tissues of *Dryophytes chrysoscelis*: (A) Immunoblots from muscle, liver, bladder, stomach, kidney, dorsal skin, ventral skin, and red blood cells are shown. A band just under the 34 kDa marker is observed in all tissues except the red blood cells. Other bands appear in various tissues, including a 55–95 kDa band that shows strongly in the liver and ventral skin. (B) Densitometry of immunoblot including all immunoreactive bands normalized to *β*actin and taken as a ratio of arbitrary units over the average warm‐acclimated tissue (mean fold change). Error bars are ±SEM. * indicates a condition with a *P* < 0.05 compared by one‐way ANOVA to data from the warm‐acclimated condition.

The pattern of protein bands in Western blots for HC‐9 varied among tissues. A band just under 34 kDa, coinciding with the predicted molecular weight of 33.69 kDa, was evident in protein extracted from muscle, liver, bladder, stomach, kidney, dorsal skin, and ventral skin in warm‐acclimated, cold‐acclimated, frozen, and thawed conditions (Fig. 4A). Red blood cells and fat (not shown) were the only tissues with no immunoreactive band in any condition. An additional ~43 kDa band appeared in the liver. A higher band, between 55 kDa and 95 kDa, is visible in the ventral skin and liver. All these bands were fully blocked by preincubation of the antibody with its antigenic peptide, indicating their specificity for HC‐9 (Fig. 4B). HC‐9 protein expression was unaffected by thermal conditions in muscle, bladder, stomach, kidney, dorsal skin, and ventral skin. However, in liver, HC‐9 protein expression was elevated in frozen and thawed animals compared with warm [F (3, 8) = 8.317, *P* ≤ 0.0077].

**Figure 5:**
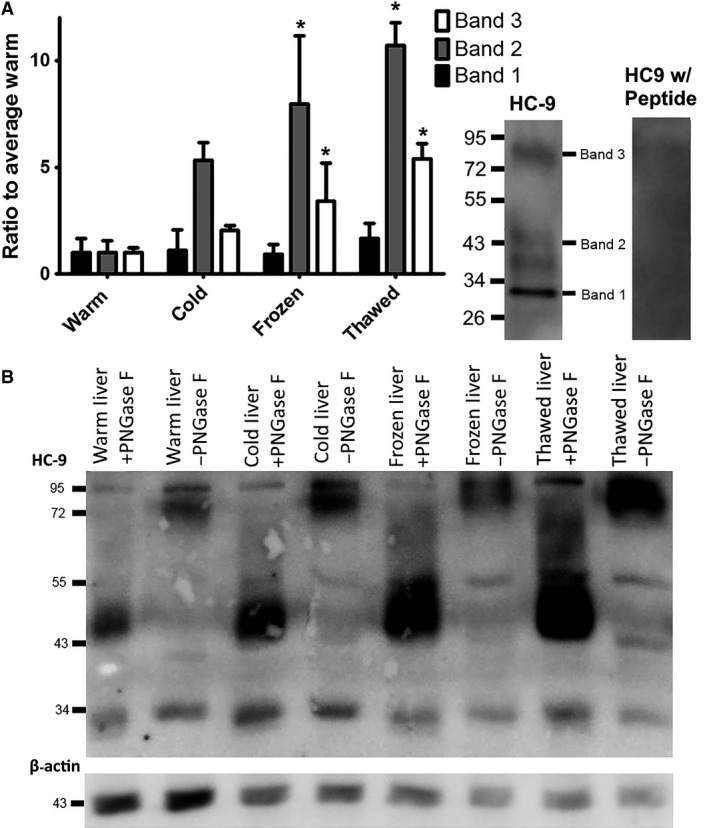
Detailed Liver Protein Expression from Immunoblots of *Dryophytes chrysoscelis* Including Treatment with PNGase F: (A) The mean measurement of the individual bands in the liver are shown by densitometry (*n* = 3). Liver showed three distinct bands. While the 33.69 kDa band did not change between warm‐acclimated, cold‐acclimated, frozen, and thawed, the other bands increase by 4‐ to 6‐fold. The error bar is ±SEM. (B) An immunoblot of the liver before and after treatment with PNGase F, a deglycosylating enzyme, across warm‐acclimated, cold‐acclimated, frozen, and thawed treefrog tissue. A band that appears around 72 kDa is reduced in intensity while a band around 45 kDa increases after treatment. * indicates a condition with a *P* < 0.05 compared by one‐way ANOVA to the band from the warm‐acclimated condition.

The 4‐ to 6‐fold increase in HC‐9 expression in frozen and thawed liver, respectively, reflected the signal from the higher MW bands but not the 34 kDa band (Fig. 5A). In particular, the 43 kDa immunoreactive band 2 increased by ~8‐ to 10‐fold over warm‐acclimated in the frozen and thawed conditions. The higher molecular weight band 3 increased by ~3‐ to 5‐fold over warm‐acclimated in the frozen and thawed conditions. After deglycosylation with PNGase F, the 55–95 kDa immunoreactive band was reduced in intensity, whereas the ~43 kDa band was enhanced (Fig. 5B), suggesting that the higher molecular weight immunoreactive band represents glycosylated protein.

### Immunolocalization of expression

**Figure 6:**
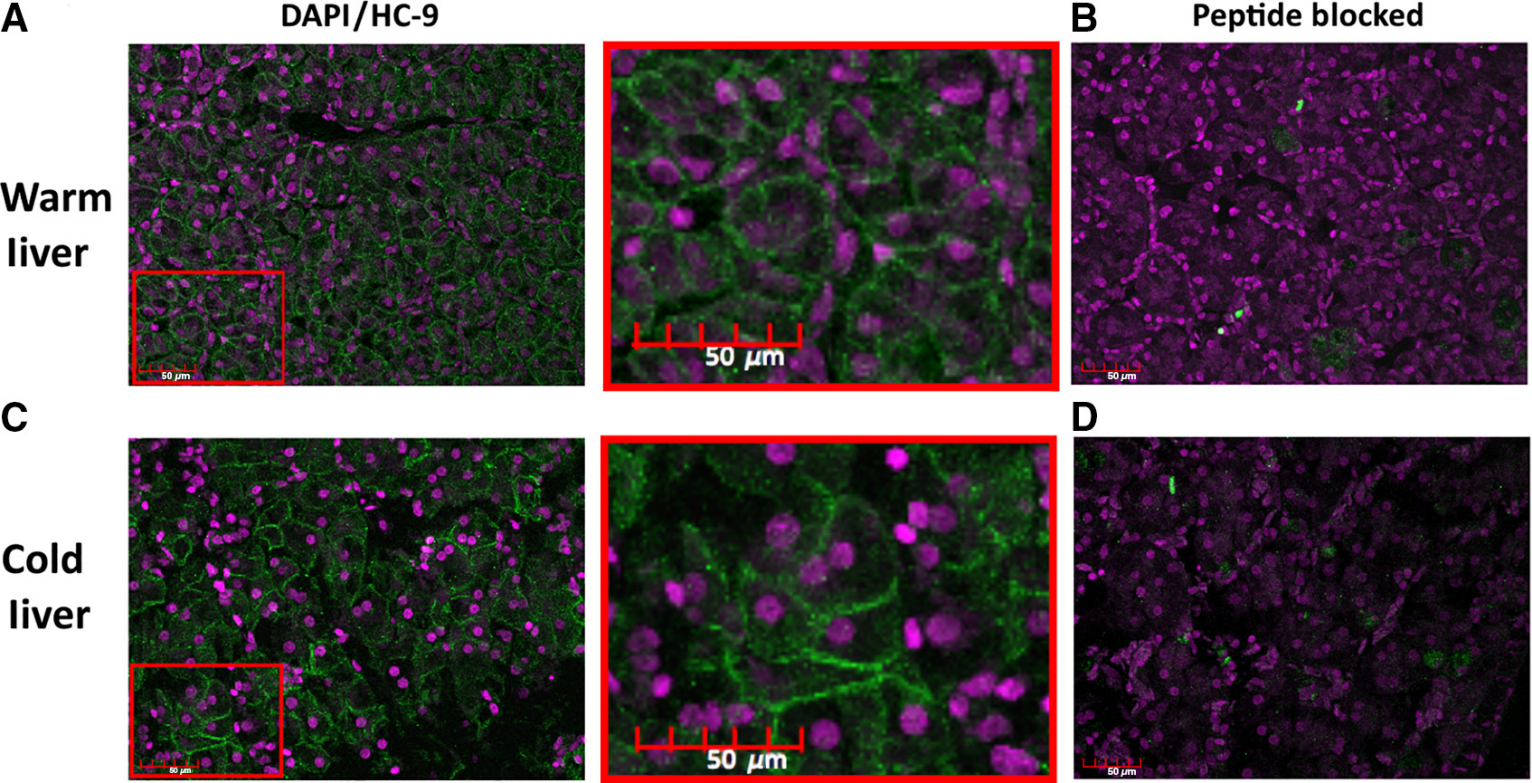
Immunofluorescence in the Liver of Warm‐acclimated and Cold‐acclimated *Dryophytes chrysoscelis*: Liver was fixed in 10 *μ*mol/L sections. Sections were stained with DAPI (purple) and HC‐9 (green) and images were taken on a confocal microscope. The DAPI stains the nucleus, whereas the HC‐9 appears in the plasma membrane of the hepatocytes in both (A) warm‐acclimated and (C) cold‐acclimated treefrog tissue. This signal is blocked after treatment with peptide in both (B) warm‐acclimated and (D) cold‐acclimated treefrog tissue.

Immunohistochemistry showed expression of HC‐9 in treefrog hepatocytes that appeared to localize to the cell membrane in liver sections from both warm‐acclimated (Fig. 6A), and cold‐acclimated (Fig. 6C) animals. The immunoreactive signal was blocked by preincubation with immunizing peptide, indicating a specificity for the HC‐9 antibody (Fig. 6B and D).

### Functional properties of HC‐9 expressed in *Xenopus* oocytes

**Figure 7:**
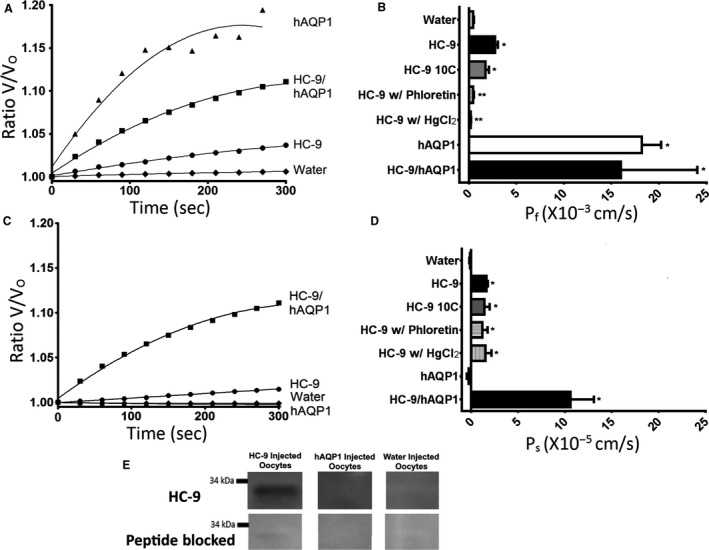
Water and Glycerol Permeability of cRNA‐injected Oocytes from Cloned HC‐9: (A) A measure of the mean volume/initial volume and (B) the mean water permeability (*P*_f_) of various injection groups (*n* = 10) given after 5 min of exposure to a hypoosmotic water solution (~67 mOsM). (C) A measure of the mean volume/initial volume over time and (D) the mean solute permeability (*P*_s_) of various injection groups (*n* = 10) after 5 min of exposure to a high glycerol (~130 mmol/L) solution. Phloretin and HgCl_2_ conditions included a 5‐min preincubation with 0.5 mmol/L phloretin or 0.3 mmol/L HgCl_2_ prior to the swelling assay. The error bar is ±SEM. (E) An immunoblot from HC‐9‐, hAQP1‐, and water‐injected oocytes incubated with HC‐9 antibody. The expected 33.69 kDa band shows only in the HC‐9‐injected oocytes, as expected. * indicates a condition with a *P* < 0.05 when compared by Student's t‐test to the *P*_f_ or *P*_s_ of the water‐injected control. ** indicates a condition with a *P* < 0.05 when compared by Student's t‐test to the P_f_ or P_s_ of HC‐9‐injected oocytes.

Oocytes injected with hAQP1, HC‐9, or HC‐9/hAQP1 showed time‐dependent osmotic swelling at room temperature compared with the water‐injected oocytes (Fig. 7A and C).

At 20°C, hAQP1‐injected oocytes rapidly swelled and occasionally burst within 300 sec after exposure to hypo‐osmotic (67 mosm/kgH_2_O) medium (Fig. 7A). For HC‐9‐injected oocytes, the calculated *P*_f_ was significantly different from water‐injected oocytes at 20°C [t (18) = 7.34, *P* ≤ 0.0001] and 10°C [t (18) = 3.374, *P* ≤ 0.0034]. By one‐way ANOVA, the *P*_f_ of HC‐9‐injected oocytes at 20°C (2.750 × 10^−3^ ± 0.9527 × 10^−3^ cm/s) significantly differed from HC‐9 injected oocytes at 10°C (1.741 × 10^−3^ ± 0.374 × 10^−3^ cm/s) with a ~37% decrease in mean *P*_f_ [F (3, 37) = 4.007, *P* ≤ 0.05]. Meanwhile, hAQP1‐injected oocytes (18.21 × 10^−3^ ± 2.010 × 10^−3^ cm/s) and dual‐injected HC‐9/hAQP1 (15.98 × 10^−3^ ± 3.042 × 10^−3^ cm/s) had a calculated *P*_f_ value 5.8‐ to 6.6‐fold greater than HC‐9, suggesting HC‐9 confers a relatively weak permeability to water (Fig. 7B). Preincubation with 0.5 mmol/L phloretin resulted in 90% reduction in calculated P_f_ of HC‐9‐injected oocytes [t (18) = 7.346, *P* ≤ 0.0001]. The effect of mercury was a similar 84% inhibition of *P*_f_ [t (16) = 7.486, *P* ≤ 0.0001; Fig. 7B].

Oocytes injected with either water or with the water‐selective hAQP1 did not swell in response to an inwardly directed glycerol gradient [t (18) = 0.77, *P* ≥ 0.4] (Fig. 7C and D). In contrast, the P_s_ of HC‐9‐injected oocytes was significantly greater than that of water‐injected oocytes at both 20°C [t (18) = 9.645, *P* ≤ 0.0001] and 10°C [t (18) = 3.828, *P* ≤ 0.0021]. Oocytes tested at 20°C (1.619 × 10^−5^ ± 0.1575 × 10^−5^ cm/s) and at 10°C (1.415 × 10^−5^ ± 0.5593 × 10^−5^ cm/s) did not differ significantly from each other (Fig. 7D). Meanwhile, the dual‐injected HC‐9/hAQP1 oocyte (10.600 × 10^−5^ ± 0.7905 × 10^−5^ cm/s) showed a 6.5‐fold increase in swelling compared to HC‐9 single injection. Preincubation with 0.5 mmol/L phloretin or 0.3 mmol/L mercury did not affect the *P*_s_ of HC‐9‐injected oocytes.

The presence of HC‐9 was confirmed in HC‐9‐injected oocytes by immunoblot. Protein from HC‐9 injected oocytes yielded the expected 33.69 kDa immunoreactive band, whereas protein from hAQP1‐injected and water‐injected oocytes did not (Fig. 7E).

## Discussion

### Structure and function of HC‐9

In this study, we examine a novel protein in Cope's gray treefrog that is homologous to the previously characterized aquaglyceroporin, AQP9. Aquaporins from the major intrinsic protein family (MIP) are typically characterized by a structure that includes six transmembrane domains, two NPA motifs, and the C‐terminus and N‐terminus residing intracellularly (Verkman et al. 2014). The two NPA motifs create a hemipore that facilitates water permeability (Verkman et al. 2014). Aquaglyceroporins possess a larger pore which in turn allows the permeability of small, neutral solutes like glycerol and urea (Jensen et al. 2001). All these features are part of the predicted secondary and tertiary structures of HC‐9.

HC‐9 differs slightly from the recently sequenced AQP‐h9 of *Dryophytes japonicus* (Hirota et al. 2015). In contrast to HC‐9, *Dryophytes japonicus’* AQP9 homolog begins at the second in‐frame AUG codon resulting in a protein that is 20 amino acids shorter. Both the *Dryophytes japonicus* and *Dryophytes chrysoscelis* genes include a similar untranslated sequence downstream of the stop codon, which adds ~780 bp to the mRNA sequence. To date, the untranslated tail shows homology only to *Xenopus tropicalis* (XM_002937673) and *Dryophytes japonicus* (LC008216.1), suggesting this feature may be unique to amphibians.

The permeability properties of HC‐9 expressed in *Xenopus* oocytes conferred substantial selectivity for glycerol over water. HC‐9 expression increased water permeability, P_f_, just modestly compared with hAQP1. However, whereas expression of HC‐9 by itself induced just modest swelling in oocytes exposed to an isosmotic glycerol gradient, that swelling increased 6.5‐fold when coexpression with hAQP1 provided a water flux pathway. These findings are similar to those of Carbrey et al. (2003) for AQP9. In contrast, AQP‐h9 from *Dryophytes japonicus* induced significant swelling of oocytes in isotonic glycerol solution (Hirota et al. 2015), suggesting they have a greater capacity to facilitate water permeability.

As for many aquaporins, including AQP9 (Tsukaguchi et al. 1998; Hirota et al. 2015), the water permeability of HC‐9 from *D. chrysoscelis* was substantially (>80%) inhibited by preincubation with mercury (0.3 mmol/L HgCl_2_). Water permeability of HC‐9 was similarly inhibited, ~80%, by 0.5 mmol/L phloretin, similar to the effect of that compound on human AQP9 (Tsukaguchi et al. 1998).

In contrast, glycerol uptake by oocytes expressing HC‐9 was not diminished by preincubation in HgCl_2_ or phloretin. This finding contrasts with data from *Dryophytes japonicus* (Hirota et al. 2015) and human (Tsukaguchi et al. 1998) AQP9 homologs, in both of which glycerol permeability was mercury‐sensitive, with 50–60% reduction in P_s_. Given the high homology of the HC‐9 sequence to AQP‐h9, this finding is unexpected. When preincubated with 0.1 mmol/L phloretin, the permeability of hAQP9 was inhibited by ~35% (Tsukaguchi et al. 1998).

The function of aquaporins in the membrane of an ectotherm, including their potential contribution to osmoregulation during cold acclimation and freezing in amphibians, depends on the effect of temperature on the functional properties of proteins. In plants, some aquaporins may be effectively gated by cold temperature, such that permeability is substantially reduced in the cold (Lee et al. 2005, 2012). Such gating has not been described for vertebrate GLPs. Changes in temperature could still exert an effect on aquaporin function either directly (via influence on protein conformation) or indirectly (via an effect of changing phospholipid composition and properties on the function of integral proteins). In the few studies that examine the effect of temperature on aquaporin function in vertebrates (Zimmerman et al. 2007) and invertebrates (Kaufmann et al. 2005), a modest diminution of permeability has been seen at a lower temperature in some cases, but not all. In this study, we were able to detect a difference in permeability to water but not glycerol at 20°C versus 10°C for oocytes expressing HC‐9. Further testing would be required to confirm HC‐9 function at different temperatures in the native membrane phospholipids from warm‐ versus cold‐acclimated treefrogs.

### Expression of HC‐9: patterns and regulation

HC‐9 mRNA was expressed across a wide range of tissues (muscle, liver, bladder, stomach, kidney, dorsal skin, ventral skin, small intestine, large intestine, brain, heart, fat, eye, lung, and red blood cells) in both warm‐acclimated and cold‐acclimated *D. chrysoscelis*. This wide distribution of expression is consistent with the pattern for AQP9 in another amphibian (*Dryophytes japonicus*; Hirota et al. 2015), as well as in mammals (Rojek et al. 2008; Lindskog et al. 2016) and some (rainbow smelt; Hall et al. 2015) but not all (zebrafish; Hamdi et al. 2009) fishes.

Despite detection of AQP9 mRNA widely across tissues, the expression of AQP9 protein may be more tissue selective. In *D. chrysoscelis*, most tissues—but not red blood cells or fat—that expressed HC‐9 mRNA also had detectable levels of HC‐9 protein, as determined by immunoblot. However, localization of HC‐9 expression in *D. chrysoscelis* has been confirmed with immunofluorescence only in hepatocytes. In *Dryophytes japonicus,* AQP‐h9 protein expression was reported for whole liver and muscle (Hirota et al. 2015), but immunofluorescence images suggest that the signal derives from erythrocytes trapped within hepatic vessels, not from hepatocytes. In mammals, AQP9 protein expression has been reported in liver, skeletal muscle, fat, brain, testis, and skin (Nicchia et al. 2001; Badaut and Regli 2004; Matsuzaki et al. 2004; Rojek et al. 2008; Inoue et al. 2009), but an assessment of immunohistological staining patterns in human tissues found AQP9 protein expression predominately in hepatocytes, with little to no conclusive expression among the other 82 cell types analyzed (Lindskog et al. 2016).

We hypothesized that AQP9 expression would increase in response to cold in *D. chrysoscelis*, associated with the accumulation of cryoprotective glycerol, and that this pattern would be particularly evident in liver, given the central role of that organ in glycerol metabolism and distribution. Our findings only partly support those predictions. In contrast to our hypothesis, mRNA expression was, on average, reduced in most tissues in cold conditions compared with warm (significantly in stomach and dorsal skin), and HC‐9 protein expression was largely unchanged by the transition to cold. Consistent with our hypotheses, however, the pattern of HC‐9 expression in liver was unique. mRNA expression in that tissue was undiminished in frozen and thawed conditions compared with cold, and protein expression was significantly elevated. Similarly, in another vertebrate that accumulates glycerol in the cold, the rainbow smelt (*Osmerus mordax)*, cold acclimation induced a decrease in mRNA expression (by qRT‐PCR) in the spleen, red blood cells, gill, brain, and intestine, but not in liver (Hall et al. 2015). These findings support the conclusion that the liver has a unique role in vertebrate ectotherms that accumulation and/or distribute glycerol during cold exposure.

The role for AQP9 must be considered within the context of other GLPs that might also be expressed. In *D. chrysoscelis*, erythrocytes express the aquaglyceroporin HC‐3 (Goldstein et al. 2010; Mutyam et al. 2011a, 2011b) but not HC‐9 protein. Conversely, in *H. japonicus,* Hirota et al. (2015) suggested that there is no signal from AQP3 (AQP‐h3BL) in erythrocytes, but AQP‐h9 was expressed. A similar phenomenon of trade‐off in the expression of aquaglyceroporins may occur in mammals; some species, e.g., rats (Roudier et al. 1998), express AQP3 in erythrocytes (but not AQP9), whereas others, for example, mice, express AQP9 but not AQP3 (Liu et al. 2007). These patterns suggest that, to some extent, the functional contributions of the several aquaglyceroporins might be interchangeable, and expression patterns may evolve differently even in closely related species.

Various tissues showed a high MW band between 5 and 95 kDa. In the warm‐acclimated ventral skin, it appeared intermittently and did not achieve statistical significance from cold, frozen, or thawed conditions. However, HC‐9 in the liver of *D. chrysoscelis* included a high MW band containing glycosylated protein, and this fraction increased during freezing and thawing. In erythrocytes from the same species, cold acclimation induced an increase in glycosylation of the AQP3 homolog HC‐3 (Mutyam et al. 2011a). N‐linked glycosylation occurs either co or posttranslationally in the endoplasmic reticulum, linking to an asparagine residue (Roth et al. 2012). In plants, N‐glycosylation of proteins is thought to be an important aspect of freeze tolerance. N‐glycosylation may be involved in protein localization, or activity (Lerouge et al. 1998; Barrero‐Gil and Salinas 2013) and may help stabilize protein structure in the cold (Buck et al. 2004; Oberg et al. 2011). In cells expressing AQP2, glycosylation is involved in sorting and trafficking the aquaporin from the Golgi apparatus to the plasma membrane (Hendriks et al. 2004). We suggest that these consequences of glycosylation may contribute to the function of GLPs in cold tolerance in *D. chrysoscelis*.

At the same time, it is noteworthy that treatment of HC‐9 from treefrog liver with PNGase F did not reduce MW to the nominal mass of 34 kDa band, but rather to 43 kDa. This suggests that a large portion of glycosylated HC‐9 has additional structural modifications such as phosphorylation or a strongly bonded dimer (Fernandez‐Llama et al. 2000; Philip et al. 2008). Although a recent model of cellular regulation of AQP9 expression focuses largely on transcriptional regulation (Lebeck 2014), our results suggest that other mechanisms, potentially including posttranslational protein modification and trafficking to the plasma membrane, are also important contributors to functional expression of these proteins.

### Role of HC‐9 in freeze tolerance

*Dryophytes chrysoscelis* and its close relative *Dryophytes versicolor* are unusual among freeze‐tolerant vertebrates in that they accumulate glycerol during cold acclimation in anticipation of freezing. Glycerol may have functions prior to freezing (e.g., to enhance supercooling capacity) as well as during freezing (stabilizing vicinal water and contributing to osmotic gradients that influence water fluxes and cell volume when ice forms). These functions depend on the ability of glycerol to cross cell membranes where most glycerol transport occurs via aquaglyceroporins.

For AQP9 to fulfill that role during transitions from warm to cold conditions, two main criteria must be satisfied. First, AQP9 must function as a glycerol pathway across a range of temperatures. Our data suggest that this is the case. At least within the context of the membrane of *Xenopus* oocytes, HC‐9 expression increases the P_s_ at both 20°C and 10°C. Second, AQP9 must be present in the membrane during those physiological states. We have currently assessed this second requirement only in the liver. In that tissue, we find what appears to be robust membrane expression in hepatocytes in both warm‐acclimated and cold‐acclimated animals.

The pattern of expression of HC‐9 in the liver is unique, including the extent of glycosylation and other posttranslational modification and the expression during freezing and thawing that is enhanced over the cold condition. In mammals, AQP9 expression in the liver is triggered by starvation and facilitates uptake of glycerol that is used for gluconeogenesis (Carbrey et al. 2003; Calamita et al. 2012). In gray treefrogs, freezing removes liquid water, thereby elevating the extracellular glycerol concentration. Upon thawing, that high extracellular glycerol, along with elevated HC‐9 expression, may allow *D. chrysoscelis* hepatocytes to take up glycerol to support the restoration of hepatic carbohydrate stores. In this model, HC‐9 in *D. chrysoscelis* hepatocytes may function similarly to AQP9 in mammalian hepatocytes, facilitating uptake of extracellular glycerol.

## Conflicts of Interest

None declared.
